# Detection of Antimicrobial Resistant *Salmonella enterica* Strains in Larval and Adult Forms of Lesser Mealworm (*Alphitobius diaperinus*) From Industrial Poultry Farms

**DOI:** 10.3389/fvets.2020.577848

**Published:** 2020-10-09

**Authors:** Alvaro Donoso, Natalia Paredes, Patricio Retamal

**Affiliations:** Laboratorio de Enfermedades Infecciosas, Departamento de Medicina Preventiva Animal, Facultad de Ciencias Veterinarias y Pecuarias, Universidad de Chile, Santiago, Chile

**Keywords:** poultry, Chile, *Salmonella*, *Alphitobius diaperinus*, drug resistance

## Abstract

The lesser mealworms (*Alphitobius diaperinus*) constitute a common cosmopolitan pest in poultry flocks and may colonize the litter in adult and larval forms. Previous studies have documented their potential as carriers of enteric pathogens. In this context, *S. enterica* constitutes a prioritized zoonotic agent in the poultry industry due to the sanitary risks and economic losses associated with its presence. The aim of this study is to describe the presence of *S. enterica* strains in larval and adult forms of *A. diaperinus* collected from poultry litter belonging to industrial farms located in the central zone of Chile. A total of 403 specimens (203 adults and 200 larvae) were sampled from three farms and 25 flocks. For bacteriological isolation, beetles were processed to differentiate external and internal contamination. Then, isolates were serotyped according to the Kauffman-White scheme and antimicrobial resistance phenotypes were determined using the disk diffusion method. Gene sequences from the megaplasmid pESI were identified through a PCR based test. These procedures led to the detection of 15 *S. enterica* isolates, belonging to serotypes Infantis (14) and Livingstone (1), from both adults (6) and larval (9) specimens, with a similar external (7) and internal (8) distribution. Furthermore, all *S*. Infantis isolates showed antimicrobial resistance and evidence of megaplasmid pESI carriage, with all possessing multidrug-resistant phenotypes. Our results confirm that *A. diaperinus* constitutes a potential reservoir of zoonotic *Salmonella* strains of sanitary and economic concern for the industry and for public health.

## Introduction

The lesser mealworms (*Alphitobius diaperinus*) constitute a common pest in poultry flocks (1), characterized as a scavenger arthropod which colonizes the litter in adult and larval forms. They are able to survive within flocks by consuming feces, food and dead birds, but can also affect residential areas in close proximity to fields treated with manure (2). This insect has been reported to serve as a vector for several enteropathogens, including *E. coli, Campylobacter*, and *Salmonella enterica*, among others (3).

*S. enterica* is an enteric pathogen that is widely distributed in nature and produces a variety of diseases in a range of hosts, including humans, mammals, birds, and reptiles. In addition, insects, plants, and unicellular organisms may also harbor bacteria in the environment (4), leading to the ubiquity and persistence of these bacteria in infecting hosts. More than 2,600 serotypes within the *S. enterica* species have been described, including both host-restricted and host-generalist serotypes (5).

The zoonotic risk of *Salmonella* is mainly associated with its transmission through consumption of contaminated animal and plant-derived foods (6). This usually results in a self-limiting gastroenteritis, although patients with some risk factors, such as infants, immunocompromised individuals and the elderly, can develop extra-intestinal infections that can cause meningitis, sepsis, and even death. In recent years, infection with this bacterium in humans has been among the most common causes of notifiable outbreaks (7). Globally, *S. enterica* serotype Enteritidis (*S. Enteritidis*) and *S. Typhimurium* represent the most common serotypes that cause disease in humans (8). However, several other bacteria, which may be classified as emergent clones or serotypes, have also been responsible for outbreaks in recent years (9, 10). In Chile, the public health service has developed food chain surveillance and food-associated outbreak investigations programs. Together these activities have established Salmonella as the most common pathogen involved in foodborne disease outbreaks with Enteritidis, Infantis, and Typhimurium the most frequently detected serotypes (11). Furthermore, the official veterinary service controls a biosafety program in the poultry industry, with specific indications for prevention and early notification of biological agents, and an official microbiological control program for exported animal products (12).

The progressive increase of antimicrobial resistant bacteria presents a current menace (13), and is cataloged by WHO as one of the most important global threats to public health. For this reason, antimicrobials have been categorized and prioritized in order to preserve their effectiveness (14). In recent years, a gradual increase in drug resistant *Salmonella* strains has been documented in the human food chain, leading to more serious clinical cases and more hospitalizations (15). The purpose of this study is to report and characterize the isolation of antimicrobial-resistant *Salmonella* serotypes in larval and adult forms of *A. diaperinus* collected from poultry litter belonging to industrial farms located in the central zone of Chile.

## Methods

### Samples

During December 2018, a total of 403 specimens (203 adults and 200 larvae) were sampled from 25 flocks belonging to three industrial farms located in the central zone of Chile. The insects were collected independently from manure and then stored in sterile 10 mL tubes.

### Bacteriological Isolation and Serotyping

Once at the lab, each sample was processed using a two-step procedure for bacteriological isolation in order to differentiate between external and internal contamination. In the first step, the insects were immersed for 10 s in 5 mL of sterile buffered peptone water (Difco BPW broth, Beckton Dicknson, Franklin lakes, NJ, USA) supplemented with 20 μg/mL of novobiocin (Sigma, St. Louis, MO, USA). In the second step, insects were recovered with tweezers and immersed for 1 min in 95% ethanol, air dried and washed with PBS, and homogenized in 1.5 mL tubes using plastic stems. Insect remains were then inoculated into 5 mL of sterile BPW broth supplemented with 20 μg/mL novobiocin. Each inoculate was incubated for 24 h at 37°C. Then 100 uL of each suspension was inoculated into modified semisolid Rappaport Vassiliadis basal medium (Oxoid, Sao Paulo, Brazil) supplemented with 20 μg/mL of novobiocin and incubated at 45.1°C for either 24 or 48 h, depending on whether or not bacterial growth was observed. Cultures were plated onto Xilose Lysine Deoxicholate agar (Difco XLD, broth, Beckton Dicknson, Franklin lakes, NJ, USA) and suspicious colonies were identified using biochemical tests and *invA* gene detection by PCR (16), using the *S*. Enteritidis SARB 16 as a control strain. Finally, *S. enterica* isolates were serotyped according to the Kauffman-White scheme (5).

### Disk Diffusion Method

Antimicrobial resistance phenotypes were determined by the disk diffusion method according to the standards recommended by the Clinical Laboratory Standards Institute (17). The following antimicrobials were evaluated: ampicillin (10 μg), amoxicillin + clavulanic acid (20/10 μg), ceftiofur (30 μg), ceftazidime (30 μg); ceftriaxone (30 μg), cefadroxil (30 μg), gentamicin (10 μg), streptomycin (10 μg), azithromycin (15 μg), tetracycline (30 μg), ciprofloxacin (5 μg), enrofloxacin (10 μg), nalidixic acid (30 μg), sulfamethoxazole + trimethoprim (20/5 μg), sulfisoxazole (10 μg), chloramphenicol (30 μg), and fosfomycin (20 μg). *Escherichia coli* ATCC 25922 was used as a control strain. The multi-drug resistance (MDR) condition was determined by the simultaneous resistance to three or more antimicrobial classes (18).

### PCR Assays

After bacterial growth was observed, nucleic acids were extracted using the DNA extraction kit (Roche®) according to the manufacturer's instructions. Then, a PCR based test was performed under standard conditions for the identification of the pESI (plasmid for emerging *S*. Infantis) genes *faeAB, ipfA, merA, pemK, ccdAB*, and *traC*, using primers described previously (19).

### Statistical Analyses

Sampling variables such as the presence of *Salmonella* in poultry flocks, the bacterial location in the *A. diaperinus* body and its developmental stage, were contrasted with isolation results through a logistic regression analysis, using the INFOSTAT (2010v) software.

## Results

Out of the samples analyzed, 10 flocks belonging to the three farms were found to be infected. A total of 15 *S. enterica* isolates were detected from lesser mealworms, including *S*. Infantis (14) and *S*. Linvingstone (1) serotypes. Additionally, all *S*. Infantis isolates showed multi-drug resistance phenotypes, and the pESI sequences were variably detected in most of the strains, with the exception of *S*. Livingstone (Table 1).

**Table 1:** Description of *Salmonella* isolates detected in lesser mealworms.

***Salmonella* serotype**	Host data^*^			Antimicrobial resistance^**^																	**pESI genes**					
	**ID**	**Loc**	**Stage**	AMP^***^	AMC^***^	EFT^***^	**CAZ**	CRO^***^	**CFR**	CN^***^	**S**	AZN^***^	**TE**	CIP^***^	ENR^***^	NA^***^	**STX**	**SF**	**C**	FOT^***^	***faeAB***	***ipfA***	***merA***	***pemK***	***ccdAB***	***traC***
Infantis	3	Int	Adult																							
Infantis	38	Ext	Adult																							
Infantis	50	Ext	Adult																							
Infantis	82	Int	Adult																							
Infantis	90	Int	Larva																							
Infantis	100	Int	Larva																							
Infantis	102	Int	Larva																							
Infantis	124	Ext	Larva																							
Infantis	126	Ext	Larva																							
Infantis	126	Int	Larva																							
Infantis	268	Ext	Larva																							
Infantis	294	Ext	Adult																							
Infantis	394	Int	Adult																							
Infantis	403	Int	Larva																							
Livingstone	356	Ext	Larva																							

* *ID, identification; Loc, location; Int, Internal; Ext, External. Gray spaces represent a phenotype or gene detection*.

** *AMP, Ampicillin; AMC, Amoxicillin/ Clavulanic acid; EFT, Ceftiofur; CAZ, Ceftazidime, CRO, Ceftriaxone; CFR, Cefadroxil; CN, Gentamicin; S, Streptomycin; AZN, Azithromycin; TE, Tetracycline; CIP, Ciprofloxacin; ENR, enrofloxacin; NA, Nalidixic acid; SXT, Sulfamethoxazole /Trimethoprim; SF, Sulfisoxazole; C, Chloramphenicol; FOT, Fosfomycin*.

*** *Critically important antimicrobials (14)*.

Analysis of infection status of flocks, bacterial location and the stage of host development variables, determined that none of them were statistically associated with *Salmonella* detection (*p* > 0.05).

## Discussion

Within poultry farms, *S. enterica* contaminates productive units and the food chain through diverse transmission pathways, including environmental sources (20) that can hold bacteria, allowing repeated infection of hosts. In fact, when such reservoirs remain unnoticed, continuous exposure and outbreaks over several years have been documented, both in animal and human settings (21, 22). This study suggests that one such potential reservoir are lesser mealworms, which are common arthropods that live on the manure within poultry facilities (23). On sampled insects, bacterial isolates were indistinctly detected both externally and internally and in larval and adult forms (*p* > 0.05). Although larvae may have a higher capacity to transmit infection to chickens than do the adult forms (23), our results suggest that beetles always present a risk of carrying and spreading *Salmonella* within poultry flock environments, that apparently depends on the bacterial dose to which these animals are exposed (24). It is feasible that *A. diaperinus* directly and indirectly transmits *Salmonella* to animals, since it is consumed by broiler chicks (25, 26) and also disseminates bacteria to the chicken manure (24).

Whether *A. diaperinus* is a reservoir host or simply a mechanical vector of *Salmonella* is an still unknown condition that new studies should address. Whatever the role, the field evidence suggests that this arthropod can survive cleaning and disinfection procedures, which presents a risk for its transmission in poultry pens (24, 27, 28). Furthermore, this insect represents a good protein source for human consumption (29), resulting in an additional public health risk if zoonotic pathogens colonize its body from the environment or through its diet (29).

It has been determined that beetles harboring *Salmonella* in their gut can shed bacteria thorough their feces for an average of 8 days, allowing persistent pathogen dispersal between flock rotations (30). In the sampled farms, routine biosecurity management practices currently incorporate an exhaustive cleaning procedure in which manure and organic matter are removed with pressurized water, and a sanitation procedure in which disinfectants are applied to pens during 14-days empty periods. Despite of these procedures, beetles have not been eradicated and persist in consecutive flocks, as does *S. enterica*, suggesting that insects play a role in the continuous exposure of birds to this bacterium. Personnel from the farms recognize the presence of small cracks and crevices within facilities, in which arthropods may survive and continuously contaminate the surrounding environment.

Regular surveillance is performed by the same farms to detect *Salmonella* infection in poultry and flocks are classified according to their infection status with this bacterium. However, in this study such condition was not a predictor of *Salmonella* detection in beetles (*p* > 0.05), suggesting that a more stringent surveillance sampling is needed, or that a differential risk exists in contamination of arthropods and chickens within pens. In fact, it has been reported that insects may be early indicators of *Salmonella* infection in flocks, with higher detection rates than other samples obtained from these environments (27).

In analyzed specimens, *S*. Infantis and *S*. Livingstone serotypes were detected. *S*. Infantis is an emerging serotype within the poultry industry, which apparently emerged 75 years ago and then expanded globally during industrialization of livestock production (31). S. Livingstone is a wide host range serotype associated with diverse hosts, including cattle (32), pigs (33), poultry (34), and sea lions (35), among others, although it is less frequently linked to disease in humans than is *S*. Infantis (8). The lower frequency of this serotype (and the absence of others) in sampled beetles may be explained by a competitive exclusion phenomenon that characterizes the transmission and colonization of *Salmonella* in poultry (36) and inside the gut of *A. diaperinus* (29).

Despite of causing milder clinical outcomes in humans than other serotypes (37), emergent *S*. Infantis strains have been associated with the acquisition of chromosomal mutations and the transmission of genetic traits, such as plasmids, which confer MDR phenotypes in most of the strains recently isolated from poultry around the globe (38–40). In Chile, antibiotics used in animals account for 95% of all antibiotics imported by the country (41), suggesting that the practice of veterinary medicine could have major impacts on the selection of drug resistant bacteria (15). In 2018, the surveillance of non-typhoidal *Salmonella* carried out by the public health service reported the emergence of *S* Infantis, as the second most frequent serotype in both intestinal and extraintestinal clinical cases, after *S*. Enteritidis. Moreover, in the same year *S*. Infantis showed the highest antimicrobial resistance levels against sulfamethoxazole /trimethoprim, chloramphenicol and ampicillin, with resistance levels ranging between 48 and 58%. In contrast, high susceptibility to ciprofloxacin was still observed, although some extraintestinal isolates (3/18) expressed resistance against this drug (11). In general, such results agree with the phenotypes observed in this study, suggesting that strains detected in lesser mealworms have been subjected to similar selection pressure within poultry environments and belong to the same transmission chains that cause disease in humans.

The *S*. Infantis drug resistance has been associated with the unique pESI megaplasmid which was initially described in Israeli isolates in 2007 (42), and along withwith some pESI-like variants, has since been described in other territories across the world (43–45). This mobile genetic structure can be transferred to other commensal or pathogenic bacteria within the host intestinal environments (19). Although contains conserved and polymorphic segments, the plasmid-associated pattern of resistance includes antimicrobials such as tetracycline, sulfametoxazole and trimethoprim, among others (19, 43), which have been subjected to positive selection and spreading of drug resistance as a result of widespread and common use (31). In this study, we found resistances against these same and other antimicrobials, as well as genetic evidence of the pESI presence, with some polymorphisms among bacterial isolates (Table 1). The existence of is plasmid might explain the MDR phenotype observed in all *S*. Infantis isolates, which is likely dispersed within local productive farms. Furthermore, these MDR phenotypes have also been associated with enhanced resistance to heavy metals and environmental fitness of the strains in which they are present (46). These characteristics represent bacterial survival mechanisms that challenge the strategies implemented by producers and sanitary authorities to control and prevent salmonellosis. The emergence of *S*. Infantis strains harboring pESI or pESI-like plasmids is a risk to public health and requires exhaustive epidemiological characterizations of the animal and environmental transmission chains so that effective control methods can be implemented.

This study has some limitations. All samples were taken during a single month and belong to industrial farms from the same company, and therefore may not be representative of the epidemiological conditions of other seasons, environments or farms throughout the country. A more extended sampling scheme, involving the collection of samples over a longer period of time and from a wider variety of companies in Chile, would have resulted in a more complete understanding of the *S*. Infantis-*A. diaperinus* relationship. In addition, a higher resolution method is needed for the plasmid description, in order to characterize and compare the pESI structure of Chilean isolates with those reported elsewhere, and for to elucidate virulence functions and risk potentials of these strains. A strength of this study is that the isolation procedure was able to discriminate between internal and external *Salmonella* contamination in adults and larvae from *A. diaperinus*, confirming the ability of this insect for bacterial transmission within flock environments.

In conclusion, there are MDR *Salmonella* strains in lesser mealworms within industrial poultry farms from Chile. These arthropods constitute a host reservoir of this zoonotic pathogen and represent economic and sanitary risks to the food chain of the country. In this regard, this study supports actions for permanent control strategies of *A. diaperinus* populations in animal facilities.

## Data Availability Statement

The raw data supporting the conclusions of this article will be made available by the authors, without undue reservation.

## Author Contributions

AD: field sampling, laboratory work, and analyses of results. NP: field sampling, analysis of results, and writing the manuscript. PR: conception, design of the study, analysis, interpretation of results, and writing the manuscript. All authors contributed to the article and approved the submitted version.

## Conflict of Interest

The authors declare that the research was conducted in the absence of any commercial or financial relationships that could be construed as a potential conflict of interest.

## References

[1] Rueda LM Axtell RC Arthropods in litter of poultry (broiler chicken and turkey) houses. J Agric Entomol. (1997) 14:81–91.

[2] Kaufman PE Reasor C Waldron JK Rutz DA. Suppression of adult lesser mealworm (*Coleoptera: Tenebrionidae*) using soil incorporation of poultry manure. J Econ Entomol. (2005) 98:1739–43. 10.1093/jee/98.5.1739 16334348 PMC7109963

[3] Hazeleger WC Bolder NM Beumer RR Jacobs-Reitsma WF. Darkling beetles (*Alphitobius diaperinus*) and their larvae as potential vectors for the transfer of *Campylobacter jejuni* and *Salmonella enterica* serovar paratyphi B variant Java between successive broiler flocks. Appl Environ Microbiol. (2008) 74:6887–91. 10.1128/AEM.00451-08 18791034 PMC2583492

[4] Gourabathini P Brandl MT Redding KS Gunderson JH Berk SG. Interactions between food-borne pathogens and protozoa isolated from lettuce and spinach. Appl Environ Microbiol. (2008) 74:2518–25. 10.1128/AEM.02709-07 18310421 PMC2293137

[5] Grimont PA Weill FX Antigenic Formulae of the *Salmonella* Serovars. 9th ed Paris: WHO Collaborating Center for Reference and Research on Salmonella, Institut Pasteur (2007). Available online at: https://www.pasteur.fr/sites/default/files/veng_0.pdf (accessed September 21, 2020).

[6] Jackson BR Griffin PM Cole D Walsh KA Chai SJ. Outbreak-associated *Salmonella enterica* serotypes and food Commodities, United States, 1998–2008. Emerg Infect Dis. (2013) 19:1239–44. 10.3201/eid1908.121511 23876503 PMC3739514

[7] Smith KF Goldberg M Rosenthal S Carlson L Chen J Chen C. Global rise in human infectious disease outbreaks. J R Soc Interface. (2014) 11:20140950. 10.1098/rsif.2014.0950 25401184 PMC4223919

[8] Hendriksen RS Vieira AR Karlsmose S Lo Fo Wong DM Jensen AB Wegener HC. Global monitoring of *Salmonella* serovar distribution from the World Health Organization Global Foodborne Infections Network Country Data Bank: results of quality assured laboratories from 2001 to 2007. Foodborne Pathog Dis. (2011) 8:887–900. 10.1089/fpd.2010.0787 21492021

[9] Penha Filho RAC Ferreira JC Kanashiro AMI Berchieri Junior A Darini A Emergent multidrug-resistant non-typhoidal *Salmonella* serovars isolated from poultry in Brazil coharboring blaCTX-M-2 and qnrB or blaCMY-2 in large plasmids. Diagn Microbiol Infect Dis. (2019) 95:93–8. 10.1016/j.diagmicrobio.2019.04.003 31221507

[10] Hindermann D Gopinath G Chase H Negrete F Althaus D Zurfluh K. *Salmonella enterica* serovar infantis from food and human infections, Switzerland, 2010–2015: poultry-related multidrug resistant clones and an emerging ESBL producing clonal lineage. Front Microbiol. (2017) 8:1322. 10.3389/fmicb.2017.01322 28751886 PMC5507995

[11] Instituto de Salud Pública *Salmonella* spp. 2014–2018. In: *Bolet*í*n de Vigilancia de Laboratorio* (2019). Available online at: http://www.ispch.cl/sites/default/files/Bolet%C3%ADnSalmonella-12052020A.pdf (accessed September 21, 2020).

[12] Servicio Agrícola y Ganadero Oficial Microbial Control Program. (2019). Available online at: https://www.sag.gob.cl/ambitos-de-accion/programa-de-control-microbiologico-oficial-pcm (accessed September 21, 2020).

[13] Hoelzer K Wong N Thomas J Talkington K Jungman E Coukell A. Antimicrobial drug use in food-producing animals and associated human health risks: what, and how strong, is the evidence? BMC Vet Res. (2017) 13:211. 10.1186/s12917-017-1131-3 28676125 PMC5496648

[14] WHO Critically Important Antimicrobials for Human Medicine. Geneva: Licence: CC BY-NC-SA 3.0 IGO (2019). p. 45.

[15] Nair D Venkitanarayanan K Kollanoor Johny A. Antibiotic-resistant *Salmonella* in the food supply and the potential role of antibiotic alternatives for control. Foods. (2018) 7:167. 10.3390/foods7100167 30314348 PMC6210005

[16] Malorny B Hoorfar J Bunge C Helmuth R. Multicenter validation of the analytical accuracy of *Salmonella* PCR: towards an international standard. Appl Environ Microbiol. (2003) 69:290–6. 10.1128/AEM.69.1.290-296.2003 12514007 PMC152403

[17] CLSI: Performance Standards for Antimicrobial Susceptibility Testing 30th ed. CLSI suplement M100. Wyne, PA: Clinical and Laboratory Standards Institute (2020).

[18] Retamal P Fresno M Dougnac C Gutierrez S Gornall V Vidal R. Genetic and phenotypic evidence of the *Salmonella enterica* serotype Enteritidis human-animal interface in Chile. Front Microbiol. (2015) 6:464. 10.3389/fmicb.2015.00464 26029196 PMC4432690

[19] Aviv G Rahav G Gal-Mor O. Horizontal transfer of the *Salmonella enterica* serovar infantis resistance and virulence plasmid pESI to the gut microbiota of warm-blooded hosts. mBio. (2016) 7:e01395–16. 10.1128/mBio.01395-16 27601577 PMC5013300

[20] Karacan Sever N Akan M. Molecular analysis of virulence genes of *Salmonella* Infantis isolated from chickens and turkeys. Microb Pathog. (2019) 126:199–204. 10.1016/j.micpath.2018.11.006 30403968

[21] Miller T Brockmann S Spackova M Wetzig J Frank C Pfeifer Y. Recurrent outbreaks caused by the same *Salmonella enterica* serovar Infantis clone in a German rehabilitation oncology clinic from 2002 to 2009. J Hosp Infect. (2018) 100:e233–8. 10.1016/j.jhin.2018.03.035 29614246

[22] Lapuz RR Umali DV Suzuki T Shirota K Katoh H. Comparison of the prevalence of *Salmonella* infection in layer hens from commercial layer farms with high and low rodent densities. Avian Dis. (2012) 56:29–34. 10.1637/9704-030711-Reg.1 22545525

[23] Leffer AM Kuttel J Martins LM Pedroso AC Astolfi-Ferreira CS Ferreira F. Vectorial competence of larvae and adults of *Alphitobius diaperinus* in the transmission of *Salmonella* Enteritidis in poultry. Vector Borne Zoonotic Dis. (2010) 10:481–7. 10.1089/vbz.2008.0089 19929223

[24] Crippen TL Sheffield CL Beier RC Nisbet DJ. The horizontal transfer of *Salmonella* between the lesser mealworm (*Alphitobius diaperinus*) and poultry manure. Zoonoses Public Health. (2018) 65:e23–33. 10.1111/zph.12404 28925562

[25] Despins JL Axtell RC. Feeding behavior and growth of turkey poults fed larvae of the darkling beetle, *Alphitobius diaperinus*. Poult Sci. (1994) 73:1526–33. 10.3382/ps.0731526 7816727

[26] Retamales J Vivallo F Robeson J Insects associated with chicken manure in a breeder poultry farm of Central Chile. Arch Med Vet. (2011) 43:79–83. 10.4067/S0301-732X2011000100011

[27] Wales AD Carrique-Mas JJ Rankin M Bell B Thind BB Davies RH. Review of the carriage of zoonotic bacteria by arthropods, with special reference to *Salmonella* in mites, flies and litter beetles. Zoonoses Public Health. (2010) 57:299–314. 10.1111/j.1863-2378.2008.01222.x 19486496

[28] Skov MN Spencer AG Hald B Petersen L Nauerby B Carstensen B. The role of litter beetles as potential reservoir for *Salmonella enterica* and thermophilic *Campylobacter* spp. between broiler flocks. Avian Dis. (2004) 48:9–18. 10.1637/5698 15077793

[29] Wynants E Crauwels S Verreth C Gianotten N Lievens B Claes J. Microbial dynamics during production of lesser mealworms (*Alphitobius diaperinus*) for human consumption at industrial scale. Food Microbiol. (2018) 70:181–91. 10.1016/j.fm.2017.09.012 29173626

[30] Crippen TL Zheng L Sheffield CL Tomberlin JK Beier RC Yu Z. Transient gut retention and persistence of *Salmonella* through metamorphosis in the lesser mealworm, *Alphitobius diaperinus* (*Coleoptera: Tenebrionidae*). J Appl Microbiol. (2012) 112:920–6. 10.1111/j.1365-2672.2012.05265.x 22380581

[31] Gymoese P Kiil K Torpdahl M Osterlund MT Sorensen G Olsen JE. WGS based study of the population structure of *Salmonella enterica* serovar Infantis. BMC Genom. (2019) 20:870. 10.1186/s12864-019-6260-6 31730461 PMC6858691

[32] Loiko MR de Paula CM Langone AC Rodrigues RQ Cibulski S Rodrigues Rde O. Genotypic and antimicrobial characterization of pathogenic bacteria at different stages of cattle slaughtering in southern Brazil. Meat Sci. (2016) 116:193–200. 10.1016/j.meatsci.2016.01.010 26896744

[33] Colello R Ruiz MJ Padin VM Roge AD Leotta G Padola NL. Detection and characterization of *Salmonella* serotypes in the production chain of two pig farms in Buenos Aires Province, Argentina. Front Microbiol. (2018) 9:1370. 10.3389/fmicb.2018.01370 30002649 PMC6031755

[34] Mancin M Barco L Losasso C Belluco S Cibin V Mazzucato M. *Salmonella* serovar distribution from non-human sources in Italy; results from the IT-Enter-Vet network. Vet Rec. (2018) 183:69. 10.1136/vr.104907 29980593

[35] Fresno M Barreto M Gutierrez S Dougnac C Abalos P Retamal P. Serotype-associated polymorphisms in a partial *rpoB* gene sequence of *Salmonella enterica*. Can J Microbiol. (2014) 60:177–81. 10.1139/cjm-2013-0872 24588392

[36] Yang Y Tellez G Latorre JD Ray PM Hernandez X Hargis BM. *Salmonella* excludes *Salmonella* in poultry: confirming an old paradigm using conventional and barcode-tagging approaches. Front Vet Sci. (2018) 5:101. 10.3389/fvets.2018.00101 29868621 PMC5964308

[37] Aviv G Cornelius A Davidovich M Cohen H Suwandi A Galeev A. Differences in the expression of SPI-1 genes pathogenicity and epidemiology between the emerging *Salmonella enterica* serovar Infantis and the model *Salmonella enterica* serovar Typhimurium. J Infect Dis. (2019) 220:1071–81. 10.1093/infdis/jiz235 31062854

[38] Cohen E Davidovich M Rokney A Valinsky L Rahav G Gal-Mor O. Emergence of new variants of antibiotic resistance genomic islands among multidrug-resistant *Salmonella enterica* in poultry. Environ Microbiol. (2020) 22:413–32. 10.1111/1462-2920.14858 31715658

[39] Sanchez-Salazar E Gudino ME Sevillano G Zurita J Guerrero-Lopez R Jaramillo K. Antibiotic resistance of *Salmonella* strains from layer poultry farms in central Ecuador. J Appl Microbiol. (2020) 128:1347–54. 10.1111/jam.14562 31867847

[40] Acar S Bulut E Stasiewicz MJ Soyer Y. Genome analysis of antimicrobial resistance, virulence, and plasmid presence in Turkish *Salmonella* serovar Infantis isolates. Int J Food Microbiol. (2019) 307:108275. 10.1016/j.ijfoodmicro.2019.108275 31408739

[41] Millanao A Barrientos-Schaffeld C Siegel-Tike C Tomova A Ivanova L Godfrey H. Antimicrobial resistance in Chile and The One Health paradigm: dealing with threats to human and veterinary health resulting from antimicrobial use in salmon aquaculture and the clinic. Rev Chil Infectol. (2018) 35:299–308. 10.4067/s0716-10182018000300299 30534910

[42] Aviv G Tsyba K Steck N Salmon-Divon M Cornelius A Rahav G. A unique megaplasmid contributes to stress tolerance and pathogenicity of an emergent *Salmonella enterica* serovar Infantis strain. Environ Microbiol. (2014) 16:977–94. 10.1111/1462-2920.12351 24320043

[43] Bogomazova AN Gordeeva VD Krylova EV Soltynskaya IV Davydova EE Ivanova OE. Mega-plasmid found worldwide confers multiple antimicrobial resistance in *Salmonella* Infantis of broiler origin in Russia. Int J Food Microbiol. (2019) 319:108497. 10.1016/j.ijfoodmicro.2019.108497 31927155

[44] Tate H Folster J Hsu C Chen J Hoffmann M Morales C. Comparative analysis of extended-spectrum-β-lactamase CTX-M-65-producing *Salmonella enterica* serovar infantis isolates from humans, food animals, and retail chickens in the United States. Antimicrob Agents Chemother. (2017) 61:e00488–17. 10.1128/AAC.00488-17 28483962 PMC5487606

[45] Alba P Leekitcharoenphon P Carfora V Amoruso R Cordaro G Di Matteo P. Molecular epidemiology of *Salmonella* Infantis in Europe: insights into the success of the bacterial host and its parasitic pESI-like megaplasmid. Microb Genom. (2020) 6:e000365. 10.1099/mgen.0.000365 32271142 PMC7371121

[46] Figueiredo R Card R Nunez-Garcia J Mendonça N da Silva G Anjum M. Multidrug-resistant *Salmonella enterica* isolated from food animal and foodstuff may also be less susceptible to heavy metals. Foodborne Pathog Dis. (2019) 16:166–72. 10.1089/fpd.2017.2418 30480469

